# Prospective dietary polyunsaturated fatty acid intake is associated with trajectories of fatty liver disease: an 8 year follow-up study from adolescence to young adulthood

**DOI:** 10.1007/s00394-022-02934-8

**Published:** 2022-07-03

**Authors:** Fuzhen Wan, Feng Pan, Oyekoya Ayonrinde, Leon A. Adams, Trevor A. Mori, Lawrence J. Beilin, Therese A. O’Sullivan, John K. Olynyk, Wendy H. Oddy

**Affiliations:** 1 Nutritional Epidemiology, Menzies Institute for Medical Research, University of Tasmania, Private Bag 23, Hobart, TAS 7000 Australia; 2 Medical School, The University of Western Australia, Perth, WA Australia; 3 Department of Gastroenterology and Hepatology, Fiona Stanley Hospital, Murdoch University, Perth, WA Australia; 4 Faculty of Health Sciences, Curtin University, Perth, WA Australia; 5 Department of Hepatology, Sir Charles Gairdner Hospital, Perth, WA Australia; 6 School of Medical and Health Science, Edith Cowan University, Perth, WA Australia

**Keywords:** Fatty liver, Group-based trajectory modelling, Fatty liver index, Dietary fats

## Abstract

**Background and aim:**

Dietary fat intake has long been associated with fatty liver. Our study aimed to determine the effect of dietary fats on longitudinal fatty liver index (FLI) trajectories from adolescence to young adulthood.

**Methods:**

Nine hundred eighty-five participants in the Raine Study, Perth, Western Australia, Australia, had cross-sectional assessments at ages 14, 17, 20 and 22 years, during which anthropometric measurements and blood tests were obtained. FLI trajectories were derived from the longitudinal FLI results. Dietary fat intake was measured with a semi-quantitative food frequency questionnaire at 14 years and log multinominal regression analyses were used to estimate relative risks.

**Results:**

Three FLI trajectories were identified and labelled as stable-low (79.1%, *N* = 782), low-to-high (13.9%, *N* = 132), and stable-high (7%, *N* = 71). The low-to-high group associated with an increased intake of the long-chain polyunsaturated fatty acids EPA, DPA and DHA (RR 1.27, 95% CI 1.10–1.48) relative to the stable-low group. Compared to the stable-low group, omega-6 and the ratio of omega-6 to omega-3 in the stable-high group were associated with an increased relative risk of 1.34 (95% CI 1.02–1.76) and 1.10 (95% CI 1.03–1.16), respectively.

**Conclusion:**

For those at high risk of fatty liver in early adolescence, high omega-6 fatty acid intake and a high ratio of omega-6 to omega-3 fatty acids are associated with increased risk of fatty liver. There should be caution in assuming these associations are causal due to possible undetected and underestimated confounding factors.

**Supplementary Information:**

The online version contains supplementary material available at 10.1007/s00394-022-02934-8.

## Introduction

The contribution of an unhealthy diet, including excessive fatty food, to development of fatty liver has been described over nearly two centuries [1]. Non-alcoholic fatty liver disease (NAFLD) is the most common form of fatty liver disease (FLD), with an estimated global prevalence of up to 24% [2] and causes a substantial economic burden to society [3]. The influence of rising obesity prevalence [4, 5], sedentary lifestyle [6], and unhealthy dietary patterns [7] on development of fatty liver has previously been reported in adolescents and young adults. Furthermore, dietary fats are considered to affect the pathogenesis of fatty liver [8].

Although some observational studies [9, 10] and dietary intervention studies [11–13] have attempted to clarify the cross-sectional relationship between dietary fats and fatty liver, there is no longitudinal study investigating the relationship between dietary fat intake and fatty liver occurrence and development in adolescents and young adult populations. In the Raine Study, a prospective association between the high-fat “Western dietary pattern” and ultrasound detected NAFLD in adolescents was found [7]. A feature of the western dietary pattern were foods high in fat such as take away foods, processed meats and fried potatoes; therefore, we aimed to test the hypothesis that high intake of dietary fat is prospectively associated with fatty liver in adolescents and young adults.

The primary aim of this study was to examine the association between baseline dietary fatty acids intake during early adolescence and subsequent longitudinal fatty liver trajectories as measured by fatty liver index (FLI) from adolescence to young adulthood. A non-invasive diagnostic model, such as FLI (based on waist circumference, body mass index (BMI) triglycerides and gamma-glutamyl-transferase) is considered an accurate and validated method of determining NAFLD in population-based epidemiological studies [14]. To achieve our primary aim, we identified distinct FLI trajectories from 14 to 22 years in the Raine Study and tested the association of early dietary fats in adolescents with these trajectories.

## Methods

### Study population

We utilised data from the Raine Study, a longitudinal cohort study in Perth, Western Australia that started as a randomised controlled trial to study the effects of frequent and repeated ultrasound scans on pregnancy outcomes. The background and methods of the Raine Study have previously been described [7]. Briefly, the original cohort of pregnant study participants (Gen1) was recruited between 1989 and 1992, at between 16 and 20 weeks gestation, resulting in 2868 live births. Follow-up assessments of the offspring (Gen2) cohort have been conducted approximately every 3 years. Approximately 70% of the Gen2 participants remained actively involved in the study at the 22-year follow-up. Clinical, biochemical and questionnaire data were collected from serial assessments during antenatal/perinatal stages, infancy, childhood, adolescence and adulthood [15].

**Fig. 1:**
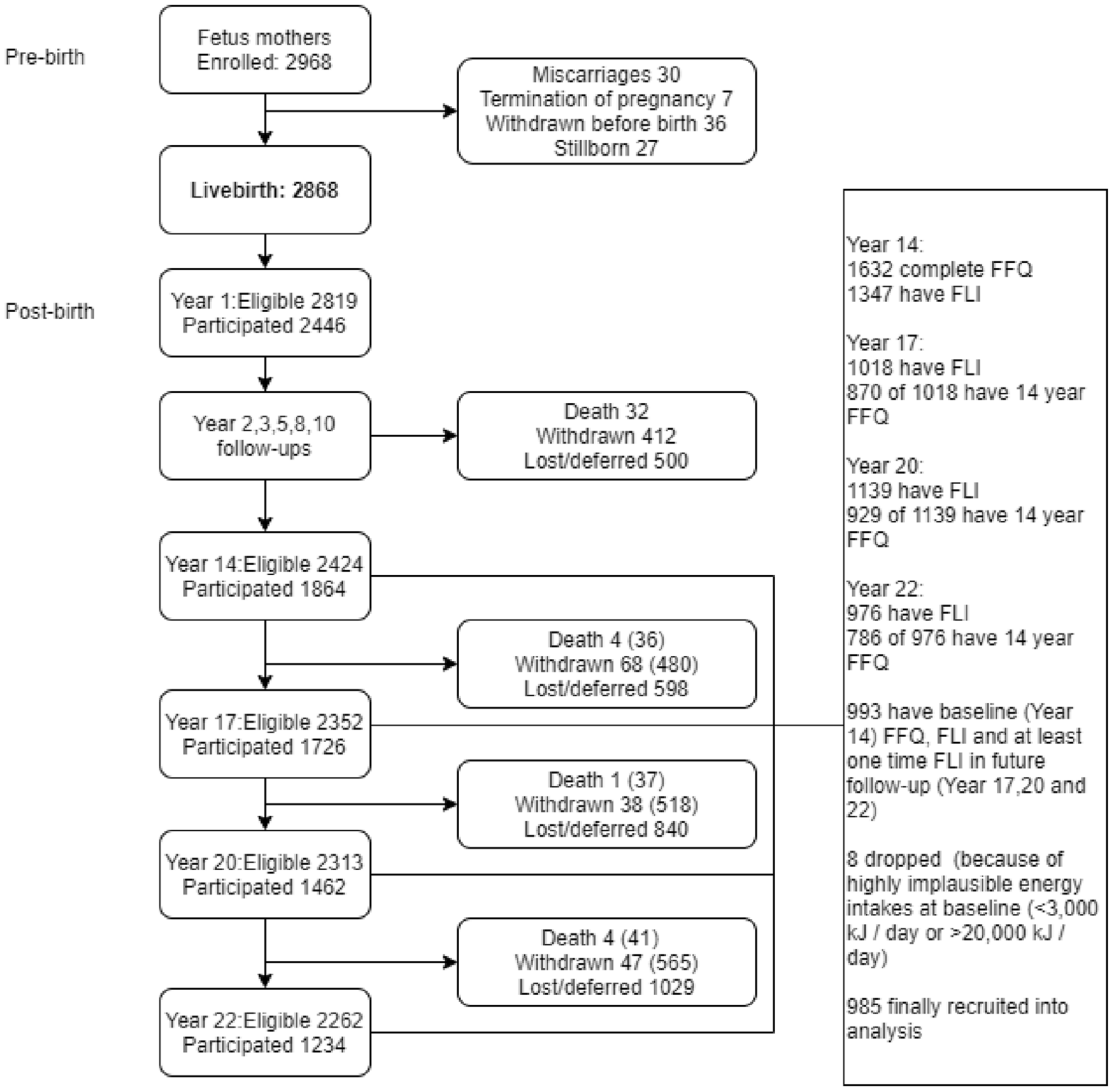
The Raine Study participant recruitment in this study detailed flowchart on participants in this study. Laboratory examination and physical measurement data from the 14, 17-, 20- and 22-year follow-up of the West Australian Pregnancy Cohort (Raine) Study are used to identify distinct FLI trajectories

Laboratory examination and physical measurement data from the 14-, 17-, 20- and 22-year follow-ups of the cohort were used in this study (see Fig. 1 for detailed information on participant recruitment).

Institutional ethics committee approval was obtained from the University of Western Australia Human Research Ethics Committee. Signed informed parental or primary carer consent during Gen1 pregnancy, Gen2 childhood and adolescence, and subsequently by Gen2 adolescents and young adults were obtained before participation in each assessment.

### Dietary fat intake assessment

A dietary fat intake assessment at 14 years was obtained from a validated semi-quantitative food frequency questionnaire (FFQ-14) developed by the Commonwealth Scientific and Industrial Research Organisation (CSIRO) in Adelaide, Australia, as previously described [16] and evaluated [17]. The parents or primary carer completed the FFQ-14 as being representative of usual dietary and nutrient intake in the previous 12 months [18]. The study nurse checked FFQ-14 responses during the clinical follow-up to clarify responses. All data from the FFQ-14 were verified by CSIRO twice, and Australian food composition data were applied to obtain estimates of usual food and nutrient intakes [19]. These included estimates of macro and micronutrients, including specific dietary fats [18] (TFA, total fatty acids; SFA, saturated fatty acids; PUFA, polyunsaturated fatty acids; MUFA, monounsaturated fatty acids; total omega-3 fatty acids (including alpha-linolenic acid and long-chain *n*–3), *n*–3; total omega-6 fatty acids (including gamma-linolenic acid and linoleic acid), *n*–6; long chain omega-3 PUFA (Eicosapentaenoic Acid, EPA + Docosapentaenoic Acid, DPA + Docosahexaenoic Acid, DHA), *n*–3 LCPUFA; and the ratio of total *n*–6 to total *n*–3 fatty acids, *n*–6:*n*–3). The FFQ-14 did not collect dietary fish oil supplement intake.

### Dietary misreporting

Dietary misreporting can contribute to measurement error in the analysis of diet–disease relationships [20]. Potential dietary misreporting in the Raine study was determined using the Goldberg method [21], which estimates the cut-offs for plausible reporting based on energy intake relative to basal metabolic rate. These cut-off values classify study participants as under-, plausible- or over-reporters of dietary intake. The method has been used widely to identify misreporting from dietary surveys and studies [22]. Because dietary underreporting may be strongly associated with the risk of overweight [23], we considered excluding under-reporters. However, excluding under-reporters removes participants at the highest risk, reducing our sample size considerably. We, therefore, created a categorical variable for misreporting where all participant data were included in the analysis. This categorical variable for misreporting was included as a covariate in our regression models. Similarly, when we summarise participants’ dietary fat intake at baseline, there is a significant difference in the proportion of dietary misreporting between trajectory groups. Therefore, we show the dietary data for plausible reporters only in Table 2 and data of the entire cohort with all categories of dietary reporting included in supplementary Table 3.

### Anthropometric and laboratory measurements

At all years, a trained research assistant weighed and measured participants in light clothing for height and weight using a calibrated stadiometer and electronic chair scales. Body mass index (BMI = weight (kg)/square of the height (m^2^)) was calculated, and subjects were categorised as underweight, normal weight, overweight and obese using the International Obesity Task Force (IOTF) criteria at 14 years [24, 25]. Blood was taken by a phlebotomist at the home of the participants from an antecubital vein after an overnight fast. Laboratory assessments were performed in the PathWest Laboratories, Perth, for serum glucose, insulin, alanine transaminase (ALT), aspartate aminotransferase (AST), gamma-glutamyl transferase (GGT), triglycerides (TG), total cholesterol, high-density lipoprotein cholesterol (HDL-C), low-density lipoprotein cholesterol (LDL-C), ferritin, transferrin saturation, high-sensitivity C-reactive protein (hs-CRP), adiponectin, serum glucose, serum insulin and leptin.

### Fatty liver index trajectories

**Fig. 2:**
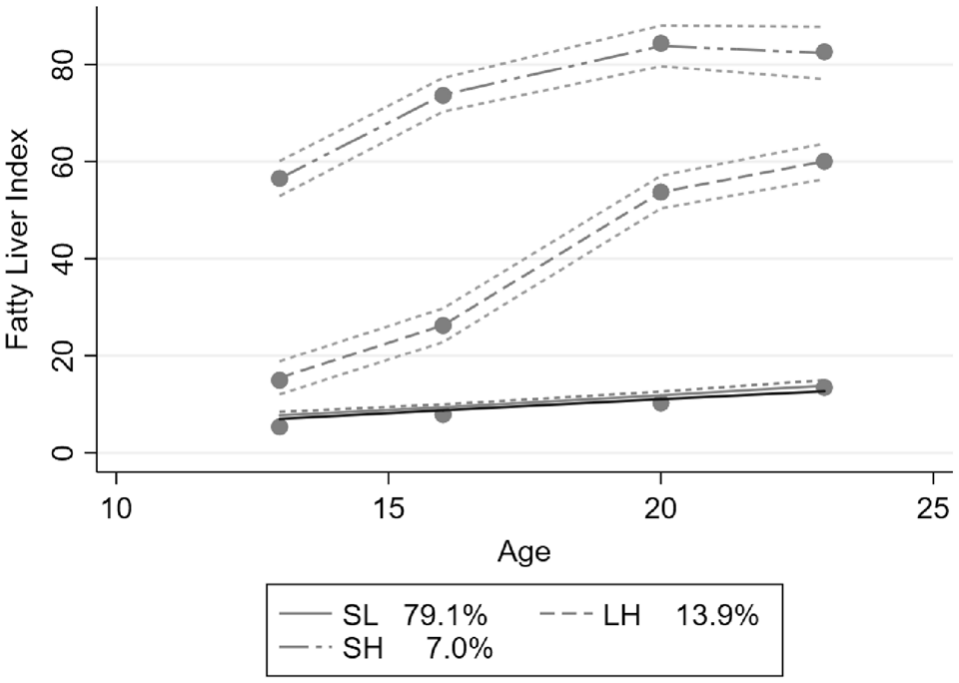
A group-based trajectory analysis of fatty liver index from 14 to 22 years (985 participants) in the Raine Study. The main dots on each trajectory represent, from left to right, the follow-up nodes from baseline (14 years) to 17, 20 and 22 years. Dotted lines either side of the trajectories are 95%CI curves. *SL* stable low risk, (*N* = 782); *LH* low risk to high risk, (*N* = 132); *SH* stable high risk, (*N* = 71)

The lack of an accurate, non-invasive, easily accessible and affordable diagnostic test is an important factor limiting research regarding fatty liver in the general population. The FLI is an algorithm based on WC, BMI, TG and GGT. The index was initially developed to detect fatty liver [26]. It is a relatively reliable tool for fatty liver research, recommended by a group of European Societies for diagnosis of fatty liver in epidemiological studies [14] and has been validated in the Raine Study cohort [27]. The FLI has been shown to have the best calibration performance compared to other prediction models attempting to identify fatty liver disease. This suggests that the FLI has a good ability to predict the risk of fatty liver among study participants at the individual level. We hypothesised that FLI is useful as a continuous variable in repeated measurements of longitudinal data to detect changes in fatty liver risk. To answer our research question, we applied a trajectory model to describe the natural history of fatty liver over eight years in Raine Study participants from adolescence (14 years) to young adulthood (22 years) using the FLI (Fig. 2).

### Covariate assessment

In the multivariate analyses of this study, there were three main types of model covariates. (1) Energy-adjusted variables, such as total energy and dietary misreporting (Model 1). (2) Demographic background variables of the cohort, such as sex, screen time and family income (Models 2 and 3). 3) Other dietary nutrient exposures and potential mediating variables included dietary patterns, total carbohydrate intake, total protein intake and homeostasis model assessment for insulin resistance (HOMA-IR) (Models 4, 5 and 6).

At each follow-up, medical status was reported, and participants with chronic diseases or use of potentially steatogenic medications (such as amiodarone and methotrexate) were excluded from the analysis. The primary caregivers of the participants were asked to report their annual family income (in Australian dollars) at the 14 year follow-up (2003–2006) and categorised as: < $ 30,000, $30,001–50,000, $50,001–78,000, and > $78,000. Parental education level and BMI were not assessed at our baseline follow-up (14 years). Also, at the 14-year follow-up, hours of physical activity per week were assessed through the International Physical Activity Questionnaire (IPAQ). Participants or their primary caregiver reported the time and frequency spent in exercising vigorously during physical education at school and outside school. Screen time, including time spent using a computer, was collected through a questionnaire with response options from ‘none at all’ to ‘4 h or more’ per day by participants’ primary caregivers. Considering that an analysis-adjusted sample using the physical activity level variable would lose much of the sample relative to the computer viewing (sedentary activity) variable, especially in some of the higher metabolic risk groups. We used the adolescent computer viewing (sedentary activity) variable for the analysis in order to obtain a larger sample with metabolic outcome. Therefore, screen time was used for the analysis to obtain a larger sample with more metabolic outcomes. The Raine Study has identified [16], evaluated [17] and applied [7] Western and healthy dietary patterns score in previous studies. In brief, these dietary pattern scores were developed using dietary data collected from the CSIRO FFQ at 14 years of age from 38 pre-defined food groups, with significant differences in fat and sugar intakes between the healthy and western dietary patterns [16]. Dietary pattern scores were adjusted as covariates in the regression models. In addition, other independent risk nutrients such as total carbohydrate (CHO) and total protein intake from the CSIRO FFQ-14 data were adjusted in the models. The HOMA-IR score was calculated as follows:

$$\begin{gathered} {\text{HOMA}} - {\text{IR score}} = {\text{ Fasting serum glucose }} \hfill \\ \,\,\,\,\,\,\,\,\,\,\,\,\,\,\,\,\,\,\,\,\,\,\,\,\,\,\,\,\,\,\,\,\,\,\,\,\,\,\,\,\,\,\,\,\,\,\,*{\text{ Fasting serum insulin}}/{22}.{5} \hfill \\ \end{gathered}$$

### Statistical analysis

Continuous descriptive data with normal distributions are presented as means ± standard deviation (SD); non-normally distributed data are reported as medians and interquartile ranges (IQRs).

Trajectories were estimated with group-based trajectory modelling (GBTM) [28]. The trajectories were derived by modelling the FLI as a function over time (participants’ age at each follow-up measurement). Data requirements for participants in the model were limited to having FLI data at the 14-year follow-up and at least one other follow-up (17, 20, or 22 years).

The best-fit model was based on the Bayesian Information Criterion (BIC) and the presence of a minimum of 5% of participants per trajectory to ensure stable estimates [28, 29]. After setting the number of trajectories and non-significant quadratic and cubic terms were removed, the best optimal model was estimated by fit indices, pragmatic evaluation, and clinical relevance. Fit indices included BIC and Akaike's information criterion (AIC), with the lower the indices, the better the model fit [28] (Supplementary Table 1). After the model was built, we tested its accuracy with the conditions suggested by Nagin [28]: the average posterior probability of assignment of each group was 0.7 or higher; the odds of correct classification was 5.0 or higher; the proportion of samples allocated to a particular group was close to the proportion estimated by the model, and the 99% confidence interval for the estimated proportion was reasonably narrow (Supplementary Table 2).

Dietary fat exposures were converted to *Z*-scores in the log multinominal regression models to obtain appropriate clinical interpretations. The total fats and sex interaction effect was not significant in our study (*P* values for interaction effects: 0.080 in low–high FLD risk group to Stable-low FLD risk group regression and 0.084 in stable-high FLD risk group to Stable-low FLD risk group regression). However, sex interaction effects of MUFA and *n*–3 dietary variables were significant in regression models for the stable-high to stable low FLD risk group (Supplementary Table 8). The conclusions of our analysis are based on the combination of the entire dataset for males and females; however, results of sex subgroup analysis are given in the supplementary material (Supplementary Table 9–15, Supplementary Figs. 1–3). Log multinominal regression analyses were conducted to estimate relative risks (RRs) and 95% confidence intervals (CIs) with multiple attributes [30] of three distinct FLI trajectories (stable-high FLD risk SH; low–high FLD risk LH) using the “stable-low FLD risk, SL” trajectory groups as a reference. RR and 95% confidence intervals (CIs) were reported for each FLI trajectory as follows: model 1 adjusted for total energy intake plus dietary misreporting; model 2 adjusted for model 1 plus sex; model 3 adjusted for model 2 plus computer viewing, and family income. We used the standard multivariate model instead of nutrient density for energy adjustment. This is because if nutrient density is used, nutrient intake will be confounded by total energy intake (in the opposite direction) as it is divided by total energy when the disease is also associated with total energy intake. BMI was not in the model because the already adjusted covariates of total energy intake, dietary misreporting and BMI were highly correlated, and additional adjustment for BMI could not be fitted to the model.

All analyses were performed using Stata 15.0 for Windows (Stata Statistical Software: College Station, Tx, USA). *P* values < 0.05 were regarded as statistically significant.

## Results

Our study population comprised 985 Gen2 adolescents aged 14 years. Participant characteristics at the 14-year follow-up are presented by different FLI trajectory groups in Table 1. The overall characteristics of the cohort showed 52% were males, 89% were of Caucasian maternal race, and 42% of the cohort had an annual family income over $70,000 at baseline.

**Table 1:** Characteristics of study population at baseline (14 years) by trajectory groups from 14 to 22 years in the Raine Study

Characteristic	Stable-low risk group	Low–high risk group	Stable-high risk group	Total
*N*	782	132	71	985
General				
Sex^†^ (proportion of males) (%)	50.1	56.8	57.8	51.6
Proportion with Caucasian mother (%)^‡^	87.6	91.7	93.0	88.5
Annual family income at baseline (%) **	*N* = 770	*N* = 131	*N* = 70	*N* = 971
< 35,000	19.5	23.7	50.7	22.4
35,000–70,000	36.8	35.9	27.9	35.9
> 70,000	43.8	40.5	21.4	41.7
Child lifestyle at 14 years				
Dietary Misreporting (%)**				
Underreporting	22.3	29.6	46.5	25.0
Plausible reporting	66.2	62.1	52.1	64.7
Overreporting	11.5	8.3	1.4	10.3
BMI (%)**				
Underweight	3.1	0	0	2.4
Healthy weight	83.6	40.0	2.8	72.0
Overweight	11.8	41.7	14.1	16.0
Obese	1.5	18.3	83.1	9.6
Physical activity (%)	*N* = 628	*N* = 102	*N* = 51	*N* = 781
Once month or less	9.7	9.8	9.8	9.7
1–3 times per week	57.2	53.9	66.7	57.4
4 + times per week	33.1	36.3	23.5	32.9
Computer viewing (%)	*N* = 778	*N* = 135	*N* = 70	*N* = 983
< 2 h per day	80.2	71.2	75.7	78.5
2–4 h per day	13.5	18.2	17.2	14.5
> 4 h per day	6.3	10.6	7.1	7.0
Healthy pattern score	− 0.01 (0.83)	0.20 (0.96)	− 0.24 (0.79)	0 (0.85)
Western pattern score	− 0.08 (0.84)	0.07 (0.83)	0.01 (0.99)	− 0.06 (0.85)
Total CHO (g/day)	274.0 (89.1)	281.5 (92.9)	261.9 (81.9)	274.2 (89.1)
Total protein (g/day)	94.1 (29.6)	100.4 (31.8)	93.1 (28.6)	94.8 (29.8)
Biochemistry (mean and standard deviation)				
ALT (U/L)**	16.19 (6.77)	17.74 (10.38)	23.06 (10.35)	16.89 (7.84)
AST (U/L)**	46.58 (2.51)	46.35 (2.67)	45.90 (2.67)	46.50 (2.52)
GGT (U/L)**	11.10 (4.31)	11.77 (3.72)	16.55 (6.75)	11.58 (4.67)
Total cholesterol (mmol/L)	4.17 (0.68)	4.09 (0.76)	4.30 (0.81)	4.17 (0.70)
Triglycerides (mmol/L)**	0.95 (0.43)	1.08 (0.45)	1.51 (1.18)	1.01 (0.54)
Insulin (mU/L)**	11.16 (7.40)	13.01 (6.27)	22.91 (14.13)	12.25 (8.49)
HDL‐C (mmol/L)**	1.44 (0.32)	1.29 (0.28)	1.13 (0.01)	1.39 (0.32)
LDL‐C (mmol/L)	2.30 (0.61)	2.31 (0.66)	2.47 (0.71)	2.31 (0.63)

*ALT* alanine transaminase; *AST* aspartate aminotransferase; *BMI* body mass index; *CHO* carbohydrates; *FFQ* food frequency questionnaire; *GGT* gamma‐glutamyl transferase; *HDL-C* high-density lipoprotein cholesterol; *LDL-C* low‐density lipoprotein cholesterol; *LH* low risk to high risk; *SH* stable high risk, *SL* stable low risk;

The results are presented as means and standard deviations except for percentages

*For *P* < 0.05 and ** for *P* < 0.01 for the difference within groups

^†^Baby’s sex was recorded at birth

^‡^Proportion with Caucasian mother data were collected at study recruitment 16–20 weeks gestation

From the 985 eligible participants, three different FLI trajectories were identified (Fig. 2) and labelled as “stable-low fatty liver risk, SL” (79.1%, *N* = 782, trajectory 1), “low-to-high fatty liver risk, LH” (13.9%, *N* = 132, trajectory 2) and “stable-high fatty liver risk, SH” (7%, *N* = 71, trajectory 3). The rising FLI trajectory indicates an increased risk of fatty liver for the individual in the SH group. The majority of participants (79%, *N* = 782) maintained a SL fatty liver risk from 14 to 22 years, while 7% (*N* = 71) and 14% (*N* = 132) were categorised in SH and LH fatty liver risk groups, respectively. For the demographic characteristics in the trajectory groups at baseline (Table 1), the SH group had the highest proportion of males (57.8%), Caucasian maternal race (93.0%) and annual family income less than $35,000 (50.7%). The SH group also had higher serum ALT, GGT, triglycerides, insulin and lower HDL-cholesterol, which may represent increased cardiometabolic risk. Lifestyle characteristics, such as dietary misreporting and BMI, were significantly different between the three trajectory groups. In the SH group, nearly 46% were under reporters as identified from the dietary misreporting analysis. The majority of subjects in the SL group (nearly 84%) were categorised as being within the healthy weight range. Progressively, the proportion of healthy weight versus overweight was similar in the LH group (40 vs 42%, respectively). Furthermore, 97% of participants in the SH group were categorised as overweight or obese. Among the laboratory biochemical indicators shown in Table 1, the SH group had the highest mean levels of all the biomarkers other than AST and HDL-C.

The baseline dietary fat intake characteristics are shown in Table 2 for plausible reporters only. Considering the group variations in energy requirements, the percentage of energy intake from specific TFA, SFA and MUFA categories of dietary fats are also reported. Energy intake from the fat categories *n*–3 LCPUFA, *n*–3 and *n*–6 are not shown because energy levels of these nutrients were very low. All indicators of fat intake characteristics (including absolute value and percentage of energy) were non-normally distributed. For most indicators, except for *n*–3 and *n*–3 LCPUFA, the SH risk group had the highest median intake of all dietary fats and percentage of energy compared to the SL and LH groups. For *n*–3 and *n*–3 LCPUFA, the LH group had the highest median intake level (total *n*–3 1.3 g/day and *n*–3 LCPUFA 288 mg/day). Except for SFA, the absolute value of most fat categories (TFA, PUFA and MUFA) showed the same trend as the percentage of energy. However, for SFA, the median absolute value of SFA in the LH group is moderate but accounts for the lowest median energy percentage (absolute value: 41.5 (g/day), energy percentage: 14.4%).

**Table 2:** FFQ dietary fat intake characteristics of participants at age 14 years presented as median and interquartile ranges (Q1, Q3) (Plausible reporters only) in the Raine Study

Dietary fats at 14 years (g/day)	Stable-low risk group	Low–high risk group	Stable-high risk group	*P* value
*N*	518	82	37	
TFA	89.9 (75.9,104.6)	98.3 (79.3,113.8)	102.1 (88.0,117.3)	0.646
Energy from TFA (%)	34.9 (31.5,38.6)	35.2 (31.7,39.0)	36.3 (32.1,39.3)	0.746
SFA	39.2 (32.3,47.2)	41.5 (32.7,49.4)	44.6 (38.1,53.3)	0.775
Energy from SFA (%)	15.3 (13.1,17.4)	14.4 (12.3,16.9)	15.5 (14.2, 17.7)	0.154
PUFA	12.6 (9.7,17.7)	16.1 (11.5,20.2)	16.8 (10.7, 20.3)	0.099
Energy from PUFA (%)	5.1 (3.8,6.3)	5.7 (4.0,7.1)	5.8 (3.9,7.0)	0.122
MUFA	31.2 (26.1,36.1)	34.8 (27.4,42.3)	34.8 (30.5,40.0)	0.551
Energy from MUFA (%)	12.1 (10.7,13.3)	12.3 (10.6,14.1)	12.4 (11.1,13.4)	0.321
*n*–3	1.2 (1.0,1.5)	1.3 (1.0,1.8)	1.2 (1.1,1.5)	0.046
*n*–3 LCPUFA (mg/day)	231.0 (164.8,306.9)	288.4 (193.3,414.9)	237.0 (167.2,301.1)	0.008
*n*–6	10.3 (7.8,14.6)	12.9 (8.8,17.2)	14.5 (9.2,17.2)	0.081
N6: N3	9.1(6.6,11.9)	9.7(6.8,12.5)	12.0(7.1,13.5)	0.015

*DHA* docosahexaenoic acid; *DPA* docosapentaenoic acid; *EPA* eicosapentaenoic acid; *n–3 LCPUFA* long-chain fatty acids (EPA + DPA + DHA); *LH* low risk to high risk; *MUFA* monounsaturated fats; *n-3* omega-3 fatty acids; *N6: N3* the ratio of total *n*–6 and total *n*–3 fatty acids; *n–6* omega-6 fatty acids; *PUFA* polyunsaturated fats; *SFA* saturated fats; *SH* stable high risk; *SL* stable low risk; *TFA* total fats

The results are presented as median and interquartile ranges (Q1, Q3)

Table 3 shows the results from multinomial analysis of the correlation between baseline dietary fats and FLI trajectories with the SL group as the reference group for each dietary fats exposure. The RR of the multinomial analyses shows *n*–3 LCPUFA and N-3: N6 at baseline (14 years) were significantly associated with the FLI trajectories after adjusting for all covariates. N-3 LCPUFA in the LH group relative to the SL group has a RR of 1.27 (95% CI 1.07–1.51) in model 5. Compared to the SL group, N-3: N6 in the SH group has RRs of 1.37 (95% CI 1.10–1.70) in model 5.

**Table 3:** Log multinomial regression models for each dietary fat (*Z*-score) at 14 years and different fatty liver index trajectories in the Raine Study (stable low risk as the reference group)

Dietary fats (*z*-score) at 14 years	Low to high (LH) risk group	*P* value	Stable high (SH) risk group	*P* value
Total fat *z*-score				
Model 1 (*N* = 985)	0.94 (0.64–1.37)	0.764	1.43 (0.80–2.55)	0.225
Model 2 (*N* = 985)	0.98 (0.68–1.43)	0.920	1.58 (0.90–2.78)	0.110
Model 3 (*N* = 966)	1.01 (0.70–1.46)	0.949	1.55 (0.90–2.65)	0.112
Model 4 (*N* = 966)	1.14 (0.74–1.78)	0.535	1.00 (0.50–2.01)	0.996
Model 5 (*N* = 966)	0.31 (0.05–2.13)	0.235	0.52 (0.02–13.90)	0.697
Model 6 (*N* = 966)	1.03 (0.72–1.48)	0.866	1.47 (0.89–2.42)	0.134
Saturated fat *z*-score				
Model 1	0.80 (0.61–1.04)	0.100	1.10 (0.74–1.63)	0.630
Model 2	0.82 (0.63–1.08)	0.158	1.19 (0.81–1.75)	0.370
Model 3	0.84 (0.65–1.11)	0.225	1.17 (0.83–1.66)	0.361
Model 4	0.89 (0.65–1.23)	0.486	0.91 (0.59–1.41)	0.674
Model 5	0.60 (0.38–0.94)	0.027	0.82 (0.45–1.50)	0.526
Model 6	0.86 (0.66–1.12)	0.267	1.16 (0.81–1.67)	0.424
Polyunsaturated fat *z*-score				
Model 1	1.14 (0.94–1.37)	0.186	1.26 (0.95–1.65)	0.107
Model 2	1.14 (0.94–1.38)	0.191	1.28 (0.96–1.71)	0.090
Model 3	1.15 (0.95–1.39)	0.164	1.20 (0.89–1.61)	0.228
Model 4	1.13 (0.934–1.36)	0.214	1.14 (0.86–1.52)	0.353
Model 5	1.12 (0.92–1.37)	0.258	1.12 (0.84–1.49)	0.448
Model 6	1.15 (0.95–1.40)	0.162	1.17 (0.88–1.55)	0.279
Monounsaturated fat *z*-score				
Model 1	1.15 (0.81–1.61)	0.438	1.44 (0.85–2.42)	0.173
Model 2	1.15 (0.82–1.60)	0.424	1.43 (0.88–2.33)	0.153
Model 3	1.16 (0.84–1.62)	0.365	1.56 (0.96–2.54)	0.074
Model 4	1.33 (0.90–1.95)	0.147	1.08 (0.56–2.08)	0.809
Model 5	1.42 (0.78–2.56)	0.249	1.48 (0.53–4.12)	0.458
Model 6	1.18 (0.85–1.63)	0.325	1.43 (0.91–2.24)	0.123
Total omega‐3 *z*-score				
Model 1	1.14 (0.97–1.33)	0.119	0.86 (0.61–1.19)	0.362
Model 2	1.14 (0.97–1.33)	0.112	0.89 (0.64–1.23)	0.482
Model 3	1.13 (0.97–1.32)	0.126	0.99 (0.71–1.38)	0.942
Model 4	1.10 (0.93–1.28)	0.263	1.00 (0.70–1.41)	0.992
Model 5	1.10 (0.93–1.31)	0.259	0.87 (0.58–1.30)	0.492
Model 6	1.13 (0.97–1.33)	0.119	0.98 (0.70–1.38)	0.918
*n*–3 LCPUFA (EPA + DPA + DHA) z-score				
Model 1	1.28 (1.11–1.47)	< 0.001	0.96 (0.73–1.25)	0.744
Model 2	1.28 (1.11–1.47)	0.001	0.91 (0.70–1.18)	0.485
Model 3	1.27 (1.10–1.48)	0.001	0.96 (0.75–1.24)	0.782
Model 4	1.21 (1.04–1.41)	0.016	0.92 (0.71–1.18)	0.517
Model 5	1.27 (1.07–1.51)	0.006	0.87 (0.65–1.17)	0.364
Model 6	1.28 (1.10–1.48)	0.001	0.99 (0.75–1.31)	0.963
Total omega‐6 z-score				
Model 1	1.10 (0.91–1.32)	0.319	1.33 (1.05–1.70)	0.019
Model 2	1.10 (0.92–1.33)	0.297	1.38 (1.08–1.78)	0.010
Model 3	1.12 (0.93–1.34)	0.240	1.34 (1.02–1.76)	0.035
Model 4	1.11 (0.93–1.33)	0.242	1.26 (0.95–1.66)	0.170
Model 5	1.10 (0.91–1.33)	0.341	1.24 (0.94–1.64)	0.130
Model 6	1.12 (0.93–1.35)	0.226	1.27 (0.99–1.64)	0.058
Total n‐6: total n:3 z-score				
Model 1	0.99 (0.94–1.03)	0.593	1.11 (1.05–1.17)	< 0.001
Model 2	0.99 (0.95–1.04)	0.641	1.12 (1.06–1.19)	< 0.001
Model 3	0.99 (0.95–1.04)	0.709	1.10 (1.03–1.16)	0.003
Model 4	1.01 (0.86–1.19)	0.900	1.35 (1.08–1.68)	0.008
Model 5	0.99 (0.83–1.17)	0.877	1.37 (1.10–1.70)	0.004
Model 6	0.97 (0.82–1.14)	0.702	1.45 (1.15–1.81)	0.001

*DHA* docosahexaenoic acid; *DPA* docosapentaenoic acid; *EPA* eicosapentaenoic acid; *HOMA-IR* homeostasis model assessment for insulin resistance; *LH* low risk to high risk; *SH* stable high risk; *SL* stable low risk

The results are presented as means and standard deviations

Model 1 adjusted for total energy + misreporting

Model 2 adjusted for Model 1 + sex

Model 3 adjusted for Model 2 + computer viewing + family income

Model 4 adjusted for Model 3 + Healthy pattern score + Western pattern score

Model 5 adjusted for Model 3 + total CHO + total protein

Model 6 adjusted for Model 3 + HOMA-IR

Supplementary Table 3 shows that the intake of dietary fats was lower in the SH group compared to that of the LH and SL groups. This may be due to the 46.5% under-reporting in the SH group (Table 1). Nevertheless, this relationship is different in *n*–3 fatty acids and *n*3 LC-PUFA, with the intake of LH group the highest, while the intake of SH group is lower than that of LH group. Supplementary Table 4 shows the results of regression analyses for the DPA, EPA, DHA, ALA and LA. In a regression model that incorporated DPA, EPA and DHA, none of the fatty acids were significantly associated with the LH group (supplementary Table 5). Supplementary Table 6 indicates the component characteristics of *n*3 LCPUFA intake (plausible reporters only). DPA had the highest median level in the LH group among the three trajectory groups.

### Sensitivity analyses

We considered the interaction of gender and the robustness of the resulting trajectory modelling (Supplementary Table 8) with the same criteria and process for males (*N* = 507) and females (*N* = 477) as a subgroup (Supplementary Tables 9 and 12). The results show that, similar to the whole population, within the subgroups, trajectories fall into three categories, i.e. a stable low-risk group, a low- to high-risk group, and a stable high risk group (Supplementary Fig. 1, 2 and 3). The relative risk of dietary exposure and trajectories were tested in the same way (Supplementary Table 11 and 15). Subgroup analyses suggest that the association between *n*–6 and N6: N3 as a population risk for the SH-SL group may be driven mostly by males (RR of *n*–6: 1.45 (1.02–2.06), RR of N6: N3: 1.16 (1.07–1.25)) (Supplementary Table 15). In addition, a risk association for monounsaturated fats in the SH-SL group was observed in the female subgroup analyses (RR of monounsaturated fats: 3.41(1.26–9.21)) (Supplementary Table 11). The *n*-3 LCPUFA as a risk for the LH group (compared to SL group) was predominantly driven by males. Furthermore, in the male and female SH groups (compared to SL group), *n*–3 LCPUFA showed contrasting effects (RR in male: 0.69 (0.48–0.97); RR in female: 1.46 (1.03–2.07)). In addition, we extended the regression analysis to other individual nutrients (e.g. sugars, dietary fibre and red meat) (Supplementary Table 16) in order to gain a more comprehensive understanding and identify potential interactions between different nutrients. After further adjustment for these nutrients the results were similar to the model results in the original manuscript, other than the effect of red meat on *n*–3 LCPUFA in the low- to high (LH) risk group (RR: 1.15 (0.97–1.38)). This may be related to the fact that red meat is a major source of n-3 LCPUFA.

## Discussion

We identified three different fatty liver index trajectories from a young population in Western Australia and observed a dietary fat intake relationship with these trajectories. The majority of participants were in the SL group, with a sustained low FLI. The SH group had FLI at a relatively persistent high level, while the LH group changed from low- to high-risk for FLD during follow-up. After controlling for total energy, dietary misreporting, sex, computer viewing and family income, intake of *n*–3 LCPUFA, *n*–6 and N6: N3 at baseline were significantly associated with prospective and longitudinal FLI trajectories over eight years of follow-up.

Our study provides a unique insight into the relationship of baseline dietary fatty acid intake on the natural history of fatty liver as assessed by trajectories of FLI from adolescence to young adulthood. We show that fatty liver development follows specific trajectories over time in young populations. FLI, as a predictor of fatty liver, has been widely used in epidemiological studies as an alternative diagnostic tool for fatty liver. The FLI incorporates BMI and our data show that adiposity is the main driver of the FLI trajectories. The prevalence of fatty liver based on FLI in 17-year-old adolescents in the Raine Study was 11.6% [27]. Meta-analyses have shown that the prevalence of FLD or NAFLD was 11.3% in children 10–14 years [31], 17.3% in adolescents 15–19 years [32] and 24% in young adults 18–35 years [33]. Our FLI trajectory data show a similar fatty liver disease trend from adolescents to young adults. These findings are worthy of highlighting given the chronicity and severity of fat accumulation in the liver is associated with subsequent risk of type 2 diabetes and cirrhosis later in life [2]. Our findings also demonstrate the importance of the transition from adolescence to young adulthood as a critical period for health promotion and health intervention to reduce the impact of chronic liver disease and cardiometabolic risk.

Our main finding is that a diet high in *n*–6 fatty acids or a high N6: N3 ratio at 14 years of age associates with an increased risk of FLD from adolescence to young adulthood. This implicates *n*–6 fatty acids as a potential risk factor in the development of FLD although this association may be influenced by dietary patterns and the intake of carbohydrates and protein in the diet. Moreover, these findings are similar to previous studies in which cross-sectional associations between N6: N3 and obesity [34] and fatty liver risk [35] were observed. In a typical Western diet, the ratio of N6: N3 is usually 15–16:1, rather than the recommended 1–4:1, which is considered the healthy range [36].

The association between N6: N3 and risk of FLD was robust in our study. Nuts and seeds are rich in *n*–6, which is why large amounts of *n*–6 can be found in refined vegetable oils such as palm oil and sunflower oil and foods cooked with these vegetable oils. Vegetable oils are a widely used source of fatty acids in the food industry for processed snacks, fast foods, cakes and cured meats that are also rich in *n*–6 and high in the ratio of N6: N3 [37]. Our study did not support saturated fatty acids as a risk factor for FLD. In the LH group, the risk of FLD was reduced if total carbohydrate and total protein were replaced with the same amount of energy from saturated fat to provide energy (Model 5). Disordered physiologic states related to the metabolic syndrome are recognised risk factors for the development of fatty liver disease in children and adults, and insulin resistance is thought to be a key pathogenic mechanism in the development of FLD [38]. Results from analyses adjusted for HOMA-IR suggest that insulin resistance may not significantly influence the association between dietary fatty acid intake and FLI trajectory.

Another interesting finding suggested here is that there could be a positive association between *n*–3 LCPUFA and liver fat. Whilst interesting, these data do not accord with clinical trials and meta-analyses that suggest *n*–3 LCPUFA may benefit fatty liver [39]. We consider this may be related to the misclassification of *n*–3 LCPUFA using FFQ data. It was noted that when using FFQ data to identify individuals with low *n*–3 PUFA intake, the FFQ was able to better capture DHA and EPA intake as opposed to ALA and total *n*–3 when compared to red blood cell or plasma fatty acid levels [40, 41]. Furthermore, dietary fatty acid nutrient intake levels and their food sources are positively correlated with body weight or BMI levels, meaning that heavier individuals consume more nutrients to maintain a balanced energy metabolism. This makes the weight factor a possible mediator in the study of nutritional epidemiology and metabolism-related outcomes. As BMI was one of the main drivers of FLI trajectories in our study, the potential positive association of fatty acids and their food sources with BMI could influence the risk of developing FLD. On the other hand, when individual intake levels were examined using the FFQ, overweight or obese individuals were more likely to under-report nutrient intake (as determined from the data shown in Table 2 and Supplementary Table 3). Thus, individuals with more severe metabolic outcomes (typically higher BMI) appear to have lower dietary intakes. We identified three trajectory groups with an increasing distribution in terms of metabolic outcomes (SH more severe than LH and LH more severe than SL) and dietary misreporting was progressive when underreporting was considered (more severe for SH than LH and more severe for LH than SL). This in turn reinforced the relative risk for the LH group as misreporting was more severe in the SH group than in the LH group. These findings suggest that the effect of n-3 LCPUFA may be overestimated, as the LH group had the highest level of n-3 LCPUFA FFQ intake level regardless of whether dietary misreporting were taken into account (Table 2 and Supplementary table 3). In addition, sensitivity analyses suggested that this association, while persisting across the many adjusted nutrient models, was no longer significant after adjustment for red meat. The association between red meat consumption and NAFLD risk is increasingly being reported [42–44] although fish and other seafood are the main dietary sources of LCPUFA. However, the 1995 National Nutrition Survey from Australia showed that close to 43% of LCPUFA intake came from meat, poultry and game products and dishes [45], and among Australian teenagers this proportion increased further to 49%, surpassing that of seafood sources (37%) [46]. The latest data from the Australian Health Survey 2011–2012 showed a similar result with the proportion of Australian adolescents consuming LCPUFA from meat, poultry and game products and dishes (33%) slightly exceeding that of seafood sources (32%) [47]. Red meat as a major source of n-3 LCPUFA is one possibility that may mediate the association found in this study. It is also important to note that the association between *n*–3 LCPUFA and FLD risk was only present in the LH risk group, which suggests heterogeneity of the association between nutrients and FLD risk in the population.

Our study validates findings in nutritional epidemiology regarding dietary misreporting, which is often associated with BMI status, educational level, and age [48, 49] and can be especially vulnerable to bias when we evaluate dietary-metabolic disease associations. Differences in dietary characteristics between plausible (Table 2) and implausible (Supplementary Table 1) populations may result from overweight and obese people under-reporting their intake of dietary fats [50].

### Strengths and limitations

To our knowledge, this is the first study to explore the association between dietary fat intake of adolescents with the longitudinal natural development of fatty liver into young adulthood. The strengths of our study include the use of data from a large cohort with prospectively collected dietary information minimising recall bias and representative of the general adolescent and young adult population in Australia. Our study focus is on the natural history of FLD risk from adolescence to young adulthood and uses an objective and validated fatty liver screening tool for longitudinal data analysis over 8 years.

Our study has several limitations. First, proper interpretation of the results of the study requires consideration of measurement errors in self-reported dietary data, unavoidable when using an FFQ [51]. We minimised this impact by applying a less biased instrument (3 day food record) and performing an internal calibration of the FFQ data [17], finding relative validity. Additionally, dietary misreporting was introduced into the analysis as a covariate factor to achieve better statistical modelling. The energy adjustment method was further applied to minimise the impact of FFQ measurement errors in statistical modelling. Second, we used FLI, a non-invasive, easily calculated but indirect marker that combines anthropometric indices and biochemical parameters to predict the risk of fatty liver disease. Although useful for large-scale epidemiological studies, it is not possible to distinguish whether any association between dietary intake and FLI is independent or mediated through body weight status (body mass index and/or waist circumference), which primarily drives FLI scores. Finally, dietary fatty acid exposure in our study was assessed only at baseline age 14 years and is strongly associated with other nutrients and beverage intake that were not assessed as potential confounders. Dietary habits during adolescence may change over time. The use of only one prospective assessment of dietary intake is a limitation of our study. We have also not accounted for degrees of physical activity.

Our study reveals that aspects of dietary fat intake are associated with the future risk of FLD in young populations. While dietary fat consumption at age 14 years is unlikely to independently lead to an increased risk of FLD 8 years later, dietary habits established during adolescence are likely to persist into adulthood, including into the reproductive years [52]. Interestingly, we examined changes in the trajectory of dietary patterns from adolescence to early adulthood (from 14, 17 to 20 years of age) in the Raine study Gen2 population [53] and found that 21% of men consuming mainly the Western Dietary Pattern Score had a stable, significant growth trajectory over time suggesting that dietary patterns established during adolescence are likely to persist into early adulthood, especially among males supporting the idea that specific populations can still benefit from early dietary interventions. Therefore, family diet education or intervention may be a viable way to prevent or reduce the rising prevalence of fatty liver among younger populations.

Future nutritional epidemiological studies should focus on the pathways by which *n*–6 fatty acids are associated with FLD, such as the mediation of inflammatory factors. Additionally, a focus on risk factors for the development of FLD is recommended, such as validating the relationships between EPA and metabolism-related outcomes in larger populations.

### Conclusions

Our study shows that the FLI trajectory from adolescence to young adulthood is a critical time for the development or continuation of fatty liver. Certain aspects of fatty acid intake in early adolescence are associated with FLI trajectories from adolescence to young adulthood. We conclude that for those at high risk of fatty liver in early adolescence, there may be potential benefits from including a lower N6:N3 fatty acid diet and reducing foods high in N6 to improve the fatty acid balance in the diet. However, great caution should be used in extrapolating the data from this observational study to dietary advice due to the many limitations outlined above.

## Supplementary Information

Below is the link to the electronic supplementary material.

Supplementary file1 (DOCX 122 KB)

## Abbreviations

AIC: Akaike’s information criterion
ALA: Alpha-linolenic acid
ALT: Alanine transaminase
AST: Aspartate aminotransferase
BMI: Body mass index
CI: Confidence interval
CHO: Carbohydrates
DHA: Docosahexaenoic acid
DPA: Docosapentaenoic acid
EPA: Eicosapentaenoic acid
FFQ: Food frequency questionnaire
FLD: Fatty liver disease
FLI: Fatty liver index
GBTM: Group-based trajectory modelling
GGT: Gamma‐glutamyl transferase
HDL-C: High-density lipoprotein cholesterol
HOMA-IR: Homeostasis model assessment for insulin resistance
LA: Linolenic acid
LDL-C: Low‐density lipoprotein cholesterol
LH: Low to high
MUFA: Monounsaturated fats
*n*–3LCPUFA: Omega -3 long-chain polyunsaturated fatty acids
*n*–3: Omega-3 fatty acids
*n*–6: Omega-6 fatty acids
N6:N3: Ratio of omega-6 and omega-3
PUFA: Polyunsaturated fats
RRs: Relative risks
SFA: Saturated fats
SH: Stable high
SL: Stable low
TFA: Total fats
TG: Triglycerides
