# Supplementary material for: Prospective dietary polyunsaturated fatty acid intake is associated with trajectories of fatty liver disease: an 8 year follow-up study from adolescence to young adulthood
Source: Eur J Nutr. 2022 Jul 3;61(8):3987–4000. doi: 10.1007/s00394-022-02934-8 (PMC9596520; doi:10.1007/s00394-022-02934-8)
Supplement: Supplementary file 1 — Supplementary file1 (DOCX 122 KB) [file 394_2022_2934_MOESM1_ESM.docx]

**Supplementary file**

**Supplementary Table 1:** AIC and BIC for FLI GBTM according to number of groups and trajectory shapes

| Number of groups | Trajectory shapes | BIC(N=3215) | BIC(N=985) | AIC |
| --- | --- | --- | --- | --- |
| 2 | 1 1 | -13371.58 | -13366.85 | -13347.28 |
| 2 | 1 2 | -13370.37 | -13366.23 | -13349.10 |
| 2 | 1 3 | -13371.58 | -13366.85 | -13347.28 |
| 2 | 2 2 | -13374.36 | -13369.63 | -13350.06 |
| 2 | 2 3 | -13375.56 | -13370.24 | -13348.22 |
| 2 | 3 3 | -13379.53 | -13373.61 | -13349.15 |
| 3 | 1 1 1 | -13031.20 | -13025.87 | -13003.86 |
| 3 | 1 2 2 | -13023.26 | -13016.76 | -12989.85 |
| 3 | 1 2 3 | -13028.64 | -13021.54 | -12992.19 |
| 3 | 1 3 3 | -13019.67 | -13011.98 | -12980.18 |
| 3 | 2 2 2 | -13027.07 | -13019.98 | -12990.62 |
| 3 | 2 2 3 | -13030.98 | -13023.29 | -12991.48 |
| 3 | 2 3 2 | -13030.46 | -13022.77 | -12990.97 |
| 3 | 2 3 3 | -13034.36 | -13026.08 | -12991.83 |
| 3 | 3 2 1 | -13039.77 | -13032.68 | -13003.32 |
| 3 | 3 2 2 | -13019.47 | -13011.78 | -12979.98 |
| 3 | 3 2 3 | -13023.50 | -13015.22 | -12980.97 |
| 3 | 3 3 2 | -13022.69 | -13014.41 | -12980.16 |
| 3 | 3 3 3 | -13026.72 | -13017.85 | -12981.15 |
| 4 | 1 1 1 1 | -12869.62 | -12862.52 | -12833.17 |
| 5 | 1 1 1 1 1 | -12769.57 | -12760.70 | -12724.00 |
| **3** | **1 3 2** | **-13015.64** | **-13008.54** | **-12979.19** |

Footnote:

AIC: Akaike Information Criterion; BIC(N=3215): Bayesian Information Criterion (for the total number of observations); BIC(N=985): Bayesian Information Criterion (for the total number of participants); FLI: fatty liver index ;GBTM: group-based trajectory modelling ;Trajectory shapes: 1= linear; 2 =quadratic; 3= cubic

The (1 3 3) and (3 2 2) model had the highest BIC compared to the other models, after further adjusting the fitted trajectories for each subgroup of specifications, (1 3 2) model was found to have a simple, comprehensible and analytical features. Both the 4 group and 5 group models gave rise to one or more groups with a tiny proportion of observations. Thus the 3-groups model with trajectories specifications (1 3 2) was selected.

**Supplementary Table 2:** Average posterior probability (AvePP) value and odds of correct classification for FLI GBTM groups

|  | FLI trajectory group | | |
| --- | --- | --- | --- |
|  | Trajectory 1 | Trajectory 2 | Trajectory 3 |
| Average posterior probability value | 0.99 | 0.93 | 0.95 |
| Odds of correct classification | 19.0 | 85.0 | 250.5 |

Footnote:

FLI: fatty liver index; GBTM: group-based trajectory modelling

**Supplementary Table 3** FFQ dietary fat intake characteristics of participants at age 14 years presented as median and interquartile ranges (Q1, Q3) (entire cohort reporting)

| Dietary fats at 14 years (g/day) | Stable-Low (SL) risk group | Low-High (LH) risk group | Stable-High (SH) risk group | P-value |
| --- | --- | --- | --- | --- |
| N | 782 | 132 | 71 |  |
| TFA | 85.6(66.1,109.0) | 89.5(67.1,112.2) | 82.3(67.0,115.6) | 0.65 |
| energy from TFA (%) | 34.9(31.3,38.5) | 34.5(30.5,38.8) | 36.6(32.1,39.6) | 0.65 |
| SFA | 37.7(27.4,48.3) | 36.3(37.9,48.6) | 35.8(27.3,48.7) | 0.77 |
| energy from SFA (%) | 15.3(13.1,17.4) | 14.4(12.3,16.9) | 15.5(14.2,17.7) | 0.09 |
| PUFA | 11.9(8.8,17.6) | 13.4 (9.3,19.1) | 15.1(9.3,18.4) | 0.10 |
| energy from PUFA (%) | 5.1(3.8,6.3) | 5.7(4.0,7.1) | 5.8(3.9,7.0) | **0.04** |
| MUFA | 29.5(22.7,37.1) | 30.3(22.8,40.5) | 28.7(22.5,36.3) | 0.55 |
| energy from MUFA (%) | 12.1(10.7,13.3) | 12.3(10.6,14.1) | 12.4(11.1,13.4) | 0.64 |
| n‐3 | 1.1(0.9,1.5) | 1.2(0.9,1.8) | 1.1(0.9,1.4) | **0.05** |
| LCPUFA (mg/day) | 221.2(151.5,295.2) | 249.2(178.4,371.3) | 210.0(146.2,299.9) | **0.01** |
| n-6 | 10.1(7.1,14.6) | 11.3(8.0,16.6) | 12.8(7.4,16.4) | 0.08 |
| N6: N3 | 8.9(6.5,11.8) | 9.3(6.2,12.0) | 11.2(6.8,14.0) | **0.01** |

Footnote:

DHA: docosahexaenoic acid; DPA: docosapentaenoic acid; EPA: eicosapentaenoic acid; LCPUFA: long-chain fatty acids (EPA + DPA + DHA); LH: low risk to high risk; MUFA: monounsaturated fats; n‐3: omega-3 fatty acids; N6: N3: the ratio of total n-6 and total n-3 fatty acids; n-6: omega-6 fatty acids; PUFA: polyunsaturated fats; SFA: saturated fats; SH: stable high risk; SL: stable low risk; TFA: total fats;

The results are presented as median and interquartile ranges (Q1, Q3).

**Supplementary Table 4** log multinomial regression models for each dietary fat (Z-score) at 14 years and different fatty liver index trajectories

| Dietary fats (z-score) at 14 years | Low to high (LH) risk group | P-value | Stable high (SH) risk group | P-value |
| --- | --- | --- | --- | --- |
| EPA (C20:5n3) z-score |  |  |  |  |
| Model 1 (N=985) | **1.27(1.10-1.46)** | **0.001** | 1.04(0.81-1.34) | 0.753 |
| Model 2 (N=985) | **1.27(1.10-1.47)** | **0.001** | 1.01(0.78-1.30) | 0.960 |
| Model 3 (N=966) | **1.27(1.09-1.49)** | **0.002** | 1.02(0.80-1.31) | 0.846 |
| DPA (C22:5n3) z-score |  |  |  |  |
| Model 1 | 1.17(1.00-1.38) | 0.056 | 1.00(0.77-1.30) | 0.983 |
| Model 2 | **1.19.(1.00-1.40)** | **0.044** | 1.01(0.78-1.30) | 0.948 |
| Model 3 | **1.18(1.00-1.39)** | **0.048** | 1.04(0.83-1.30) | 0.751 |
| DHA (C22:6n3) z-score |  |  |  |  |
| Model 1 | **1.14(1.06-1.23)** | **0.000** | 0.98(0.77-1.24) | 0.850 |
| Model 2 | **1.18(1.07-1.30)** | **0.001** | 0.92(0.70-1.20) | 0.529 |
| Model 3 | **1.17(1.06-1.30)** | **0.003** | 0.89(0.68-1.17) | 0. |
| ALA (C18:3n3) z-score |  |  |  |  |
| Model 1 | 1.04(0.87-1.23) | 0.679 | 0.87(0.63-1.19) | 0.375 |
| Model 2 | 1.06(0.89-1.26) | 0.497 | 0.92(0.67-1.26) | 0.599 |
| Model 3 | 1.06(0.90-1.25) | 0.474 | 1.04(0.75-1.44) | 0.800 |
| LA (C18:2n6) z-score |  |  |  |  |
| Model 1 | 1.11(0.92-1.33) | 0.278 | **1.36(1.06-1.74)** | **0.017** |
| Model 2 | 1.09(0.91-1.31) | 0.334 | **1.33(1.05-1.69)** | **0.020** |
| Model 3 | 1.11(0.93-1.34) | 0.258 | **1.34(1.02-1.76)** | **0.037** |

Footnote:

ALA: alpha linolenic (C18:3n3); EPA: Eicosapentaenoic Acid (C20:5n3); DPA: Docosapentaenoic Acid (C22:5n3); DHA: Docosahexaenoic Acid (C22:6n3); LA: linoleic (C18:2n6);

**: p-value <0.001

**Supplementary Table 5** multivariate log multinomial regression of dietary fats (Z-score) at 14 years and different fatty liver index trajectories in one mixed model

| Dietary fats (z-score) at 14 years | Low to high (LH) risk group | P-value | Stable high (SH) risk group | P-value |
| --- | --- | --- | --- | --- |
| EPA (C20:5n3) z-score |  |  |  |  |
| Model 1 (N=985) | 1.36(0.87-2.11) | 0.174 | 1.85(0.79-4.33) | 0.156 |
| Model 2 (N=985) | 1.19(0.75-1.88) | 0.453 | 1.58(0.69-3.64) | 0.283 |
| Model 3 (N=966) | 1.20(0.74-1.95) | 0.452 | 1.60(0.72-3.58) | 0.248 |
| DPA (C22:5n3) z-score |  |  |  |  |
| Model 1 | 0.89(0.62-1.29) | 0.541 | 0.64(0.33-1.27) | 0.202 |
| Model 2 | 1.00.(0.69-1.45) | 0.986 | 0.75(0.39-1.46) | 0.402 |
| Model 3 | 1.00(0.68-1.46) | 0.988 | 0.79(0.43-1.45) | 0.454 |
| DHA (C22:6n3) z-score |  |  |  |  |
| Model 1 | 1.01(0.82-1.25) | 0.910 | 0.74(0.45-1.20) | 0.217 |
| Model 2 | 1.08(0.86-1.36) | 0.521 | 0.73(0.45-1.19) | 0.201 |
| Model 3 | 1.07(0.84-1.36) | 0.582 | 0.68(0.41-1.12) | 0.129 |

Footnote:

EPA: Eicosapentaenoic Acid (C20:5n3); DPA: Docosapentaenoic Acid (C22:5n3); DHA: Docosahexaenoic Acid (C22:6n3);

**Supplementary Table 6** FFQ EPA, DPA and DHA intake characteristics of participants at age 14 years presented as median and interquartile ranges (Q1, Q3) (Plausible reporters only)

| Dietary fats at 14 years (mg/day) | Stable-Low (SL) risk group | Low-High (LH) risk group | Stable-High (SH) risk group | P-value |
| --- | --- | --- | --- | --- |
| N | 518 | 82 | 37 |  |
| EPA (C20:5n3) | 66.1(46.3,87.4) | 87.4(55.8,116.4) | 68.2(53.7,100.1) | **<0.01** |
| DPA (C22:5n3) | 94.3(64.8,125.8) | 107.8(72.4,157.7) | 98.1(80.0,140.3) | **0.03** |
| DHA (C22:6n3) | 63.3(39.3,95.8) | 85.8(54.1,137.7) | 59.4(41.3,87.6) | **<0.01** |

Footnote:

EPA: Eicosapentaenoic Acid (C20:5n3); DPA: Docosapentaenoic Acid (C22:5n3); DHA: Docosahexaenoic Acid (C22:6n3)

**Supplementary Table 7** Log multinomial regression models for each dietary fats (Z-score) at 14 years and different fatty liver index trajectories in the Raine Study (stable low risk as the reference group, plausible reporters only, N=637).

| Dietary fats (z-score) at 14 years | Low to high (LH) risk group | P-value | Stable high (SH) risk group | P-value |
| --- | --- | --- | --- | --- |
| Total fat z-score |  |  |  |  |
| Model 1 (N=637) | 1.00(0.64-1.59) | 0.968 | 1.23(0.61-2.46) | 0.565 |
| Model 2 (N=637) | 1.03(0.66-1.62) | 0.881 | 1.30(0.66-2.56) | 0.451 |
| Model 3 (N=627) | 1.09(0.70-1.70) | 0.698 | 1.06(0.53-2.15) | 0.866 |
| Saturated fat z-score |  |  |  |  |
| Model 1 | 0.74(0.53-1.03) | 0.078 | 1.14(0.69-1.87) | 0.616 |
| Model 2 | 0.76(0.55-1.06) | 0.105 | 1.19(0.74-1.93) | 0.469 |
| Model 3 | 0.78(0.56-1.09) | 0.147 | 1.10(0.67-1.82) | 0.707 |
| Polyunsaturated fat z-score |  |  |  |  |
| Model 1 | 1.20(0.95-1.52) | 0.131 | 1.12(0.78-1.60) | 0.554 |
| Model 2 | 1.20(0.95-1.53) | 0.130 | 1.11(0.77-1.61) | 0.0567 |
| Model 3 | 1.24(0.97-1.58) | 0.081 | 0.93(0.64-1.35) | 0.704 |
| Monounsaturated fats z-score |  |  |  |  |
| Model 1 | 1.31(0.86-1.99) | 0.205 | 1.21(0.64-2.29) | 0.556 |
| Model 2 | 1.30(0.86-1.95) | 0.211 | 1.19(0.66-2.17) | 0.566 |
| Model 3 | 1.33(0.89-1.97) | 0.166 | 1.17(0.61-2.25) | 0.636 |
| Total omega‐3 z-score |  |  |  |  |
| Model 1 | 1.10(0.88-1.37) | 0.391 | 0.78(0.49-1.24) | 0.294 |
| Model 2 | 1.10(0.89-1.36) | 0.387 | 0.79(0.51-1.24) | 0.312 |
| Model 3 | 1.10(0.89-1.37) | 0.382 | 0.87(0.52-1.45) | 0.594 |
| n-3 LCPUFA z-score |  |  |  |  |
| Model 1 | **1.42(1.24-1.63)** | **<0.001** | 0.77(0.53-1.11) | 0.164 |
| Model 2 | **1.46(1.25-1.71)** | **<0.001** | 0.77(0.53-1.11) | 0.161 |
| Model 3 | **1.48(1.25-1.76)** | **<0.001** | 0.83(0.59-1.18) | 0.309 |
| Total omega‐6 z-score |  |  |  |  |
| Model 1 | 1.17(0.93-1.47) | 0.171 | 1.21(0.85-1.71) | 0.291 |
| Model 2 | 1.18(0.94-1.48) | 0.163 | 1.21(0.86-1.71) | 0.279 |
| Model 3 | 1.22(0.97-1.53) | 0.091 | 0.99(0.68-1.45) | 0.977 |
| Total n‐6: total n:3 z-score |  |  |  |  |
| Model 1 | 1.02(0.96-1.08) | 0.503 | **1.10(1.01-1.20)** | **0.027** |
| Model 2 | 1.02(0.96-1.08) | 0.499 | **1.10(1.01-1.20)** | **0.028** |
| Model 3 | 1.03(0.97-1.09) | 0.350 | 1.04(0.95-1.15) | 0.391 |

Footnote:

DHA: docosahexaenoic acid; DPA: docosapentaenoic acid; EPA: eicosapentaenoic acid; LH: low risk to high risk; SH: stable high risk; SL: stable low risk.

The results are presented as means and standard deviations.

Model 1 adjusted for total energy; Model 2 adjusted for 1+ sex ; Model 3 adjusted for 2+ computer viewing + family income.

**Supplementary Table 8** P value of interaction’s test in all regression models

|  | LH-SL regression interaction | SH-SL regression interaction |
| --- | --- | --- |
| Total fat | 0.083 | 0.082 |
| Saturated fat | 0.098 | 0.186 |
| Polyunsaturated fat | 0.058 | 0.270 |
| Monounsaturated fat | 0.121 | **0.031** |
| Total omega‐3 | 0.065 | **0.046** |
| Total omega‐6 | 0.112 | 0.537 |
| n-3 LCPUFA (EPA + DPA +DHA) | 0.521 | **0.002** |
| Total n‐6: total n:3 | 0.975 | 0.143 |

Footnote:

DHA: docosahexaenoic acid; DPA: docosapentaenoic acid; EPA: eicosapentaenoic acid; LH: low risk to high risk; SH: stable high risk; SL: stable low risk.

**Supplementary Table 9** AIC and BIC for FLI GBTM according to number of groups and trajectory shapes in female.

| Number of groups | Trajectory shapes | BIC(N=1540) | BIC(N=477) | AIC(N=477) |
| --- | --- | --- | --- | --- |
| 2 | 1 1 | -6280.46 | -6276.94 | -6264.44 |
| 2 | 1 2 | -6279.97 | -6275.87 | -6261.28 |
| 2 | 1 3 | -6279.07 | -6274.39 | -6257.72 |
| 2 | 2 2 | -6283.58 | -6278.89 | -6262.22 |
| 2 | 2 3 | -6282.73 | -6277.45 | -6258.70 |
| 2 | 3 3 | -6285.77 | -6279.91 | -6259.07 |
| 3 | 1 1 1 | -6102.94 | -6097.67 | -6078.91 |
| 3 | 1 2 2 | -6091.26 | -6084.82 | -6061.90 |
| 3 | 1 2 3 | -6097.50 | -6090.46 | -6065.46 |
| 3 | 1 3 3 | -6089.79 | -6082.17 | -6055.08 |
| 3 | 2 2 2 | -6094.88 | -6087.85 | -6062.84 |
| 3 | 2 2 3 | -6098.19 | -6090.57 | -6063.48 |
| 3 | 2 3 2 | -6097.57 | -6089.95 | -6062.86 |
| 3 | 2 3 3 | -6093.38 | -6085.18 | -6056.00 |
| 3 | 3 2 1 | -6097.83 | -6090.80 | -6065.79 |
| 3 | 3 2 2 | -6089.98 | -6082.36 | -6055.27 |
| 3 | 3 2 3 | -6100.87 | -6092.67 | -6063.50 |
| 3 | 3 3 2 | -6092.60 | -6084.40 | -6055.22 |
| 3 | 3 3 3 | -6096.00 | -6087.21 | -6055.95 |
| 4 | 1 1 1 1 | -6017.52 | -6010.49 | -5985.48 |
| 5 | 1 1 1 1 1 | -6028.53 | -6019.74 | -5988.48 |
| **3** | **1 3 3** | **-6089.79** | **-6082.17** | **-6055.08** |

Footnote:

AIC: Akaike Information Criterion; BIC(N=1540): Bayesian Information Criterion (for the total number of observations) ;BIC(N=477): Bayesian Information Criterion (for the total number of participants); FLI: fatty liver index ;GBTM: group-based trajectory modelling,Trajectory shapes: 1= linear; 2 =quadratic; 3= cubic

**Supplementary Figure 1**: A group-based trajectory analysis of fatty liver index from 14 to 22 years (477 female participants) in the Raine Study.

Foot note:

The main dots on each trajectory represent, from left to right, the follow-up nodes from baseline (14 years) to 17, 20 and 22 years. Dotted lines either side of the trajectories are 95%CI curves. trajectory 1: N=385 ; trajectory 2: N=64 ; trajectory 3: N=28

**Supplementary Table 10** : Average posterior probability (AvePP) value and odds of correct classification for FLI GBTM female groups

|  | FLI trajectory group | | |
| --- | --- | --- | --- |
|  | Trajectory 1 | Trajectory 2 | Trajectory 3 |
| Average posterior probability value | 0.99 | 0.95 | 0.97 |
| Odds of correct classification | 165.8 | 287.3 | 1138.5 |

Footnote:

FLI: fatty liver index; GBTM: group-based trajectory modelling

**Supplementary Table 11**. Log multinomial regression models for each dietary fat (Z-score) at 14 years and different Female fatty liver index trajectories in the Raine Study female (Trajectory 1 as the reference group).

| Dietary fats (z-score) at 14 years | RR of Trajectory 2 to 1 | P-value | RR of Trajectory 3 to 1 | P-value |
| --- | --- | --- | --- | --- |
| Total fat z-score |  |  |  |  |
| Model 1 (N=477) | 0.73(0.40-1.31) | 0.288 | 2.72(0.85-8.69) | 0.090 |
| Model 2 (N=470) | 0.74(0.40-1.37) | 0.341 | 2.38(0.71-8.02) | 0.161 |
| Saturated fat z-score |  |  |  |  |
| Model 1 | **0.60(0.39-0.91)** | **0.017** | 1.32(0.60-2.89) | 0.492 |
| Model 2 | **0.55(0.34-0.86)** | **0.010** | 1.01(0.45-2.27) | 0.985 |
| Polyunsaturated fat z-score |  |  |  |  |
| Model 1 | 1.23(0.92-1.63) | 0.155 | 1.25(0.78-1.99) | 0.347 |
| Model 2 | 1.26(0.95-1.68) | 0.115 | 1.48(0.86-2.55) | 0.157 |
| Monounsaturated fats z-score |  |  |  |  |
| Model 1 | 1.00(0.55-1.83) | 0.994 | **3.41(1.26-9.21)** | **0.016** |
| Model 2 | 1.08(0.58-2.04) | 0.802 | **3.99(1.44-11.02)** | **0.008** |
| Total omega‐3 z-score |  |  |  |  |
| Model 1 | **1.27(1.01-1.60)** | **0.043** | 1.06(0.67-1.67) | 0.813 |
| Model 2 | 1.22(0.97-1.54) | 0.088 | 1.06(0.64-1.74) | 0.817 |
| long-chain fatty acids  (EPA + DPA +DHA) z-score |  |  |  |  |
| Model 1 | 1.22(0.97-1.53) | 0.090 | **1.46(1.03-2.07)** | **0.033** |
| Model 2 | 1.24(0.97-1.58) | 0.082 | 1.43(0.950-2.15) | 0.088 |
| Total omega‐6 z-score |  |  |  |  |
| Model 1 | 1.14(0.87-1.94) | 0.243 | 1.28(0.85-1.94) | 0.243 |
| Model 2 | 1.12(0.87-1.44) | 0.364 | 1.46(0.94-2.26) | 0.092 |
| Total n‐6: total n:3 z-score |  |  |  |  |
| Model 1 | 0.99(0.93-1.05) | 0.824 | 1.07(0.97-1.18) | 0.158 |
| Model 2 | 1.00(0.94-1.07) | 0.966 | 1.09(0.99-1.20) | 0.077 |

Footnote:

DHA: docosahexaenoic acid; DPA: docosapentaenoic acid; EPA: eicosapentaenoic acid;

The results are presented as means and standard deviations.

Model 1 adjusted for + misreporting + total energy; Model 2 adjusted for 2+ computer viewing + family income.

**Supplementary Table 12** AIC and BIC for FLI GBTM according to number of groups and trajectory shapes in male

| Number of groups | Trajectory shapes | BIC(N=1675) | BIC(N=508) | AIC(N=508) |
| --- | --- | --- | --- | --- |
| 2 | 1 1 | -7061.43 | -7057.85 | -7045.16 |
| 2 | 1 2 | -7060.43 | -7056.25 | -7041.45 |
| 2 | 1 3 | -7064.04 | -7059.27 | -7042.35 |
| 2 | 2 2 | -7063.83 | -7059.06 | -7042.14 |
| 2 | 2 3 | -7067.44 | -7062.07 | -7043.04 |
| 2 | 3 3 | -7071.12 | -7065.15 | -7044.00 |
| 3 | 1 1 1 | -6900.56 | -6895.19 | -6876.15 |
| 3 | 1 2 2 | -6904.10 | -6897.54 | -6874.27 |
| 3 | 1 2 3 | -6907.07 | -6899.91 | -6874.52 |
| **3** | **1 3 2** | **-6901.63** | **-6894.47** | **-6869.08** |
| 3 | 1 3 3 | -6904.93 | -6897.17 | -6869.68 |
| 3 | 2 2 2 | -6907.29 | -6900.13 | -6874.75 |
| 3 | 2 2 3 | -6910.75 | -6902.99 | -6875.50 |
| 3 | 2 3 2 | -6910.94 | -6903.18 | -6875.68 |
| 3 | 2 3 3 | -6908.04 | -6899.69 | -6870.08 |
| 3 | 3 2 1 | -6905.62 | -6898.46 | -6873.08 |
| 3 | 3 2 2 | -6904.74 | -6896.98 | -6869.48 |
| 3 | 3 2 3 | -6908.04 | -6899.69 | -6870.08 |
| 3 | 3 3 2 | -6908.25 | -6899.90 | -6870.29 |
| 3 | 3 3 3 | -6911.56 | -6902.61 | -6870.88 |
| 4 | 1 1 1 1 | -6836.38 | -6829.23 | -6803.84 |
| 4 | 1 2 1 1 | -6028.53 | -6019.74 | -5988.48 |
| 4 | 1 1 2 1 | -6850.49 | -6842.73 | -6815.23 |
| **4** | **1 1 1 2** | **-6838.27** | **-6830.52** | **-6803.02** |
| 4 | 1 2 2 1 | -6837.73 | -6829.37 | -6799.76 |
| 4 | 1 2 1 2 | -6847.16 | -6838.81 | -6809.20 |
| 5 | 1 1 1 1 1 | -6766.52 | -6757.57 | -6725.84 |

Footnote:

AIC: Akaike Information Criterion; BIC(N=3215): Bayesian Information Criterion (for the total number of observations); BIC(N=985): Bayesian Information Criterion (for the total number of participants); FLI: fatty liver index; GBTM: group-based trajectory modelling,Trajectory shapes: 1= linear; 2 =quadratic; 3= cubic

**Supplementary Figure** 2: A group-based trajectory analysis of fatty liver index from 14 to 22 years (508 male participants) in the Raine Study.

Footnote: The main dots on each trajectory represent, from left to right, the follow-up nodes from baseline (14 years) to 17, 20 and 22 years. Dotted lines either side of the trajectories are 95%CI curves. trajectory 1: N=79; trajectory 2: N=359; trajectory 3: N=42; trajectory 4: N=28

**Supplementary Table 13**: Average posterior probability (AvePP) value and odds of correct classification for FLI GBTM male groups

|  | FLI trajectory group | | |  |
| --- | --- | --- | --- | --- |
|  | Trajectory 1 | Trajectory 2 | Trajectory 3 | Trajectory 4 |
| Average posterior probability value | 0.87 | 0.96 | 0.92 | 0.99 |
| Odds of correct classification | 83.2 | 37.6 | 248.6 | 3369.2 |

**Supplementary Figure** 3: A group-based trajectory analysis of fatty liver index from 14 to 22 years (508 male participants) in the Raine Study.

Foot note:

The main dots on each trajectory represent, from left to right, the follow-up nodes from baseline (14 years) to 17, 20 and 22 years. Dotted lines either side of the trajectories are 95%CI curves. trajectory 1: N=404; trajectory 2: N=61; trajectory 3: N=43

**Supplementary Table 14:** Average posterior probability (AvePP) value and odds of correct classification for FLI GBTM male group

|  | FLI trajectory group | | |
| --- | --- | --- | --- |
|  | Trajectory 1 | Trajectory 2 | Trajectory 3 |
| Average posterior probability value | 0.99 | 0.93 | 0.98 |
| Odds of correct classification | 71.1 | 194.5 | 1024.1 |

Considering the clinical relevance, there is no huge difference in explain the clinical meaning between 4 groups and 3 groups, and the following regression analysis will be conducted with 3 groups as an example.

**Supplementary Table 15** Log multinomial regression models for each dietary fat (Z-score) at 14 years and different Male fatty liver index trajectories in the Raine Study (stable low risk as the reference group).

（based on Trajectory shapes（1 3 2））

| Dietary fats (z-score) at 14 years | RR of Trajectory 2 to 1 | P-value | RR of Trajectory 3 to 1 | P-value |
| --- | --- | --- | --- | --- |
| Total fat z-score |  |  |  |  |
| Model 1 (N=508) | 1.10(0.63-1.92) | 0.731 | 1.12(0.59-2.12) | 0.725 |
| Model 2 (N=496) | 1.14(0.66-1.99) | 0.634 | 1.14(0.62-82.11) | 0.675 |
| Saturated fat z-score |  |  |  |  |
| Model 1 | 0.79(0.52-1.211) | 0.283 | 1.05(0.64-1.72) | 0.849 |
| Model 2 | 0.82(0.54-1.25) | 0.362 | 1.05(0.67-1.66) | 0.824 |
| Polyunsaturated fat z-score |  |  |  |  |
| Model 1 | 1.25(0.93-1.67) | 0.138 | 1.30(0.89-1.92) | 0.178 |
| Model 2 | 1.26(0.94-1.71) | 0.128 | 1.13(0.78-1.64) | 0.522 |
| Monounsaturated fats z-score |  |  |  |  |
| Model 1 | 1.16(0.72-1.88) | 0.543 | 0.99(0.58-1.71) | 0.986 |
| Model 2 | 1.19(0.73-1.93) | 0.490 | 1.15(0.66-2.00) | 0.625 |
| Total omega‐3 z-score |  |  |  |  |
| Model 1 | 0.96(0.72-1.23) | 0.803 | 0.73(0.44-1.20) | 0.216 |
| Model 2 | 0.97(0.73-1.29) | 0.829 | 0.83(0.49-1.39) | 0.473 |
| long-chain fatty acids  (EPA + DPA +DHA) z-score |  |  |  |  |
| Model 1 | **1.26(1.01-1.58)** | **0.045** | **0.69(0.48-0.97)** | **0.035** |
| Model 2 | 1.25(0.99-1.56) | 0.058 | 0.81(0.57-1.15) | 0.246 |
| Total omega‐6 z-score |  |  |  |  |
| Model 1 | 1.21(0.92-1.60) | 0.168 | **1.45(1.02-2.06)** | **0.036** |
| Model 2 | 1.23(0.93-1.64) | 0.150 | 1.29(0.90-1.85) | 0.171 |
| Total n‐6: total n:3 z-score |  |  |  |  |
| Model 1 | 1.04(0.97-1.11) | 0.295 | **1.16(1.07-1.25)** | **<0.001** |
| Model 2 | 1.04(0.97-1.11) | 0.296 | **1.11(1.02-1.21)** | **0.013** |

Footnote:

DHA: docosahexaenoic acid; DPA: docosapentaenoic acid; EPA: eicosapentaenoic acid.

The results are presented as means and standard deviations.

Model 1 adjusted for + misreporting + total energy; Model 2 adjusted for 2+ computer viewing + family income.

We considered trajectories between males and females and results comparing males and females. We found that long chain fatty acid intake was significantly protective in males in the stable high group compared to the stable low risk group, whereas these fatty acids were a risk in females in the stable high group compared to the stable low risk group. Over the entire population there was no effect in this group of long chain fatty acid intake, and omega 6 and the ratio of n6: n3 appears significant in males only. However, because the sex FLI interaction effect was not significant in our study, we base our conclusions on the entire dataset of males and females combined.

**Supplementary Table 16** Log multinomial regression models for additional individual nutrients adjusted for model (dietary fibre, total sugar intake and red meat consumption)

| Dietary fats (z-score) at 14 years | Low to high (LH) risk group | P-value | Stable high (SH) risk group | P-value |
| --- | --- | --- | --- | --- |
| Total fat z-score |  |  |  |  |
| Model 7 (N=966) | 1.13 (0.73-1.74) | 0.578 | 1.52 (0.80-2.86) | 0.199 |
| Model 8 (N=966) | 0.93 (0.57-1.53) | 0.790 | 1.10 (0.52-2.33) | 0.813 |
| Model 9 (N=966) | 0.98 (0.67-1.41) | 0.899 | 1.53 (0.87-2.69) | 0.136 |
| Saturated fat z-score |  |  |  |  |
| Model 7 | 0.86 (0.63-1.17) | 0.343 | 1.07 (0.70-1.64) | 0.759 |
| Model 8 | 0.78 (0.57-1.07) | 0.119 | 1.03 (0.68-1.58) | 0.879 |
| Model 9 | 0.82 (0.62-1.09) | 0.177 | 1.17 (0.81-1.68) | 0.405 |
| Polyunsaturated fat z-score |  |  |  |  |
| Model 7 | 1.14 (0.94-1.39) | 0.175 | 1.23 (0.92-1.64) | 0.168 |
| Model 8 | 1.15 (0.94-1.41) | 0.170 | 1.06 (0.78-1.44) | 0.719 |
| Model 9 | 1.15 (0.94-1.40) | 0.164 | 1.17 (0.87-1.58) | 0.292 |
| Monounsaturated fat z-score |  |  |  |  |
| Model 7 | 1.31 (0.91-1.88) | 0.151 | 1.50 (0.88-2.56) | 0.132 |
| Model 8 | 1.20 (0.78-1.84) | 0.412 | 1.17 (0.57-2.37) | 0.671 |
| Model 9 | 1.05 (0.75-1.45) | 0.794 | 1.55 (0.94-2.57) | 0.089 |
| Total omega‐3 z-score |  |  |  |  |
| Model 7 | 1.13 (0.96-1.32) | 0.141 | 1.01 (0.72-1.41) | 0.967 |
| Model 8 | 1.12 (0.96-1.32) | 0.157 | 0.86 (0.58-1.27) | 0.449 |
| Model 9 | 1.08 (0.92-1.28) | 0.348 | 0.95 (0.67-1.36) | 0.793 |
| n-3 LCPUFA  (EPA + DPA +DHA) z-score |  |  |  |  |
| Model 7 | **1.28 (1.10-1.48)** | **0.001** | 0.98 (0.76-1.27) | 0.904 |
| Model 8 | **1.28 (1.10-1.49)** | **0.001** | 0.86 (0.66-1.12) | 0.252 |
| Model 9 | 1.15 (0.97-1.38) | 0.107 | 0.92 (0.69-1.24) | 0.601 |
| Total omega‐6 z-score |  |  |  |  |
| Model 7 | 1.11 (0.93-1.34) | 0.249 | **1.35 (1.03-1.76)** | **0.028** |
| Model 8 | 1.11 (0.92-1.34) | 0.281 | 1.21 (0.89-1.63) | 0.220 |
| Model 9 | 1.12 (0.93-1.35) | 0.232 | 1.31 (0.99-1.73) | 0.056 |
| Total n‐6: total n:3 z-score |  |  |  |  |
| Model 7 | 0.97 (0.82-1.14) | 0.715 | **1.37 (1.12-1.69)** | **0.003** |
| Model 8 | 0.97 (0.82-1.15) | 0.741 | **1.35 (1.09-1.67)** | **0.007** |
| Model 9 | 1.03 (0.87-1.22) | 0.759 | **1.40 (1.12-1.74)** | **0.003** |

Footnote:

Model 7 adjusted for Model 3+ dietary fibre

Model 8 adjusted for Model 3+ total sugar intake

Model 9 adjusted for Model 3+ red meat consumption
